# TREM2 promotes adipogenesis of PDGFR-α^+^ adipose stem cells but is dispensable for adipose remodeling and metabolic health during diet-induced obesity

**DOI:** 10.3389/fendo.2026.1738472

**Published:** 2026-04-23

**Authors:** Anja Dobrijevic, Ana Korosec, Julia Stefanie Brunner, Lenka Matejovicova, Anna Gemza, Karin Lakovits, Hon Shing Lam, Aloña Agirre-Lizaso, Gernot Schabbauer, Sylvia Knapp, Omar Sharif

**Affiliations:** 1 Institute of Vascular Biology and Thrombosis Research, Centre for Physiology and Pharmacology, Medical University of Vienna, Vienna, Austria; 2 Christian Doppler Laboratory for Immunometabolism and Systems Biology of Obesity-Related Diseases (InSpiReD)., Vienna, Austria; 3 Research Division of Infection Biology, Department of Medicine I, Medical University of Vienna, Vienna, Austria

**Keywords:** adipogenesis, adipose stem cell, metabolic health, obesity, TREM2, white adipose tissue

## Abstract

White adipose tissue (WAT) expands through adipocyte hypertrophy or hyperplasia-associated differentiation of progenitor adipose stem cells (ASC). The triggering receptor expressed on myeloid cells 2 (TREM2) reportedly promotes adipocyte differentiation and whole body TREM2 deletion in mice leads to adipose hypertrophy and worsened metabolic health. However, whether TREM2 expression in ASC is involved is unknown. Here, we find TREM2 is expressed on platelet derived growth factor receptor-α (PDGFR-α) positive ASC in obesity. While global deletion of TREM2 strongly attenuated adipocyte differentiation in three different cell culture models, conditional TREM2 deletion within PDGFR-α^+^ ASC impacted early adipocyte differentiation. Obese animals where TREM2 was specifically deleted in PDGFR-α^+^ expressing cells exhibited moderately increased ASC, but WAT morphology, macrophage amounts as well as glucose and insulin tolerance were comparable to littermate controls. Thus, although TREM2 is important for adipocyte differentiation in cell culture, its expression in ASC is dispensable for WAT remodeling and metabolic health during obesity.

## 1 Introduction

Adipose tissue, aside from adipocytes, contains various stromal vascular fraction (SVF) cells including, but not limited to macrophages, vascular endothelial cells, adipose stem cells (ASC), and extracellular matrix proteins that are crucial for healthy adipose expansion (1–3). ASC derived from white adipose tissue (WAT) are tremendously heterogeneous and can also be referred to as fibro-adipogenic progenitors (FAPs). Those that become committed pre-adipocytes are characterized by the expression of fibroblast, mesenchymal and stem cell markers (Sca-1, CD29, PDGFR-α) and the absence of blood and endothelial cell-specific markers (CD45, CD31, Ter119, hereafter called Lin^-^) (1–3). Lineage tracing experiments conclusively demonstrate tracing of PDGFR-α^+^ ASC into adipocytes in WAT. They further demonstrate that PDGFR signaling is downregulated during ASC transition to mature adipocytes (4, 5). Further, Lin^-^CD29^+^CD34^+^Sca1^+^CD24^+^ ASC start proliferating within three days of high-fat diet (HFD) feeding with proliferation rates returning to baseline during prolonged obesity (6). CD24^+^ ASC particularly produce adipocytes *in vivo* following cell transplantation (7). Committed pre-adipocytes further express Peroxisome Proliferator-activated Receptor Gamma (PPAR-γ), the master regulator of adipocyte differentiation, that drives expression of genes involved in lipid metabolism, including, perilipin (*Plin1*), adiponectin (*Adipoq*) and fatty acid binding protein 4 (*Fabp4*) (8). WAT expansion occurs through increased ASC proliferation and differentiation which boosts adipocyte number (hyperplasia or adipogenesis) or via enlargement of existing mature adipocytes (hypertrophy).

Physiological adipose growth is associated with adipocyte proliferation and differentiation, minimal inflammation and anti-inflammatory M2 resident macrophages (1, 3, 9). Conversely, pathological adipose expansion that occurs during diet induced obesity (DIO) is connected to constrained adipogenesis, adipocyte hypertrophy, limited angiogenesis and fibrosis (1, 3, 10). Moreover, accumulation of pro-inflammatory M1 adipose tissue macrophages (ATMs) promotes an inflammatory microenvironment that contributes to insulin resistance (9, 11, 12). Exhausted adipose expansion is characterized by its inability to store lipids, resulting in ectopic lipid spillover (1, 3, 10). Thus adipose tissue has finite expandability upon caloric excess.

The triggering receptor expressed on myeloid cells 2 (TREM2) is primarily expressed on various myeloid cells including microglia, osteoclasts, subsets of hepatic macrophages and ATMs (13, 14). The TREM2 gene consists of 5 exons encoding a protein of 230 amino acids containing; a signal peptide (encoded by exon 1), extracellular immunoglobulin-like V-type domain, a stalk region (both encoded by exons 2/3), a helical transmembrane domain (encoded by exon 4) and a short cytoplasmic tail lacking signaling motifs (encoded by exon 5) (13, 14). The extracellular domain of TREM2 binds various ligands, most notably lipids and apoptotic cells, and is shed by the proteases ADAM10 and ADAM17 (15–17). This generates sTREM2 which may prevent further activation of TREM2 signaling that occurs through the adaptor proteins DAP12/DAP10 (13, 14).

In obesity, TREM2 is crucial for healthy adipose expansion and remodeling. Obese mice globally devoid of TREM2 in exons 3 and 4 (hereafter *Trem*2^-/-^ mice) exhibit augmented adipose hypertrophy and worsened metabolic health (18–20). Like obese *Trem*2^-/-^ animals, obese mice overexpressing TREM2 exhibit worsened metabolic health. This is attributed to a promotion in adipogenesis by TREM2 as blockage of TREM2 signaling via sTREM2 attenuates differentiation of 3T3L1 fibroblasts into adipocytes. Further, while no differences in weight occur between obese *Trem*2^-/-^ animals and controls, mice overexpressing TREM2 exhibit increased adiposity (21). Since TREM2 overexpression may mediate off target effects and sTREM2 might have pro-survival effects as well as possibly interact with other unknown receptors (22, 23), the relevance of ASC-expressed TREM2 during adipocyte differentiation and its contributions to metabolic health remains unclear.

In this study, utilizing TREM2-deficient ASC as well as conditional mouse genetics to specifically delete TREM2 in PDGFR-α^+^ cells, we examine the contributions of pre-adipocyte-expressed TREM2 to adipocyte differentiation, morphology and metabolic health.

## 2 Results

### 2.1 TREM2 is expressed in adipocyte progenitors during metabolic stress

**Figure 1:**
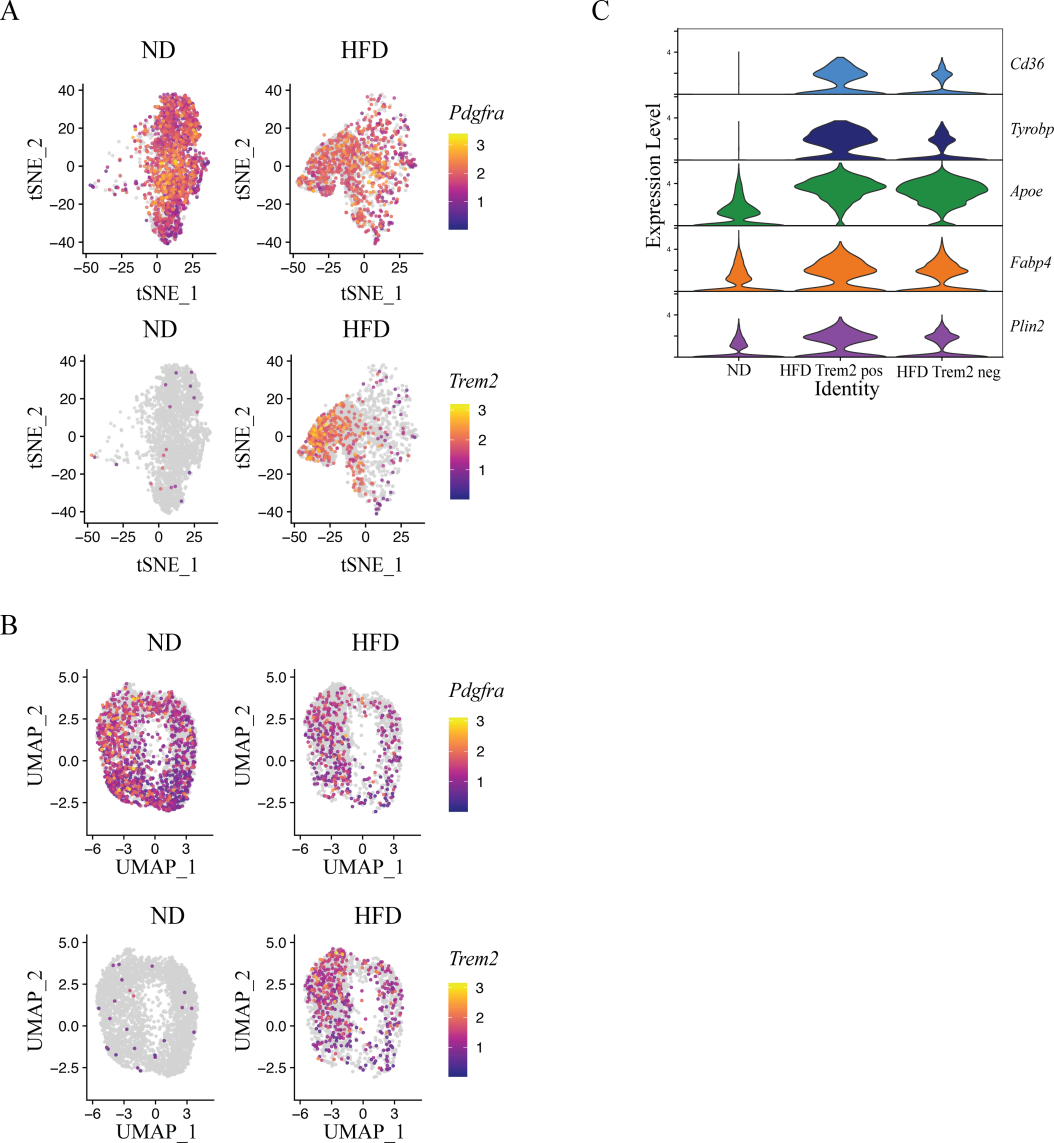
*Trem2* is induced in PDGFR-α^+^ adipocyte progenitors post high-fat diet feeding and associates with a lipid-handling gene signature. **(A)** t-distributed stochastic neighbor embedding (t-SNE) plot of ASC from eWAT of lean (18 weeks ND) and obese (12 weeks HFD) mice from Gene Expression Omnibus number GSE237143. The original clustering and cell annotations were retained. Color intensity represents normalized expression levels of *Pdgfra* (top) and *Trem2* (bottom). **(B)** Uniform Manifold Approximation and Projection (UMAP) plot of FAP from eWAT of lean (18 weeks ND) and obese (18 weeks HFD) mice from Gene Expression Omnibus number GSE160729. The original clustering and cell annotations were retained. Color intensity represents normalized expression levels of *Pdgfra* (top) and *Trem2* (bottom). **(C)** Violin plots showing scaled expression levels of the indicated genes (*Cd36, Tyrobp, Apoe, Fabp4, and Plin2*). Cells were stratified into *Trem2*-positive and *Trem2*-negative subsets based on expression profiles shown in **(A)**.

Utilizing publicly available single-cell RNA sequencing data (scRNA-seq) (24), we first examined which cell types express *Trem2* in the SVF of epididymal WAT (eWAT) in normal chow diet (ND) or HFD-fed animals, visualizing the clustered data using t-distributed Stochastic Neighbour Embedding (t-SNE) through the Seurat R package. While little *Trem2* was observed in ND fed animals, as expected *Trem2* expression increased post-HFD mostly on *Adgre1*-expressing ATMs (**Supplementary Figure 1A**). Examination of HFD ASCs in this data set revealed there was minor quantity of cells, expressing *Adgre1* (243 out of 3056 cells, 7.95%) and *Itgam* (50 out of 3056 cells, 1.64%) (**Supplementary Figure 1A**). These are likely residual doublets post-doublet removal by the original authors (24). We next selected specifically the ASC cluster and removed *Adgre1* or *Itgam* expressing single or double positive cells (285 cells out of 3056 cells, 9.33% excluded) computationally from further analysis, calculating a new t-SNE projection of ASC. This revealed *Trem2* expression in a population of ASC expressing *Pdgfra*, (494 *Trem2*^+^ cells out of 2771, 17.83%) which was specific for HFD feeding (**Figure 1A**).

These observations were confirmed in an independent data-set employing single-nucleus RNA sequencing (snRNA-seq) (25), where in addition to immune cells (*Adgre1* expressing macrophages), *Trem2* was observed in *Pdgfra* expressing FAPs, that are analogous to ASC (**Supplementary Figure 1B**). In this data-set out of 2135 FAPs in the HFD condition, 255 cells were positive for *Adgre1* (11.94%) and 124 (5.81%) for *Itgam*. We next selected specifically the FAP cluster, removing *Adgre1* or *Itgam* expressing single or double positive cells (364 out of 2135 cells, 17.05% excluded) computationally from further analysis and calculated new Uniform Manifold Approximation and Projection (UMAP). Analogous to the previous data-set, here we observed *Trem2* expression in 479 FAPs post-HFD fed (out of 1771) (**Figure 1B**). Together, these data indicated that although *Trem2* was not expressed at baseline in PDGFR-α^+^ ASC/FAPs, its expression was increased upon metabolic stress instigated by DIO.

Since TREM2 signaling that is mediated by DAP-12 (*Tyrobp*) can enhance expression of lipid uptake and sensing genes (17), we next compared expression of various lipid-associated genes in *Trem2*^+^ versus *Trem2*^-^ ASC. Compared to ND fed animals, the expression of genes associated with lipid transport (*Fabp4, Cd36*) and storage (*Plin2*), were elevated in ASC from HFD-fed animals. These genes and *Tyrobp* exhibited most expression in *Trem2*^+^ ASC, suggesting that in metabolic disease, pre-adipocyte TREM2 signaling is associated with lipid sensing, uptake and possibly adipogenesis (**Figure 1C**).

### 2.2 TREM2 enhances adipocyte differentiation

**Figure 2:**
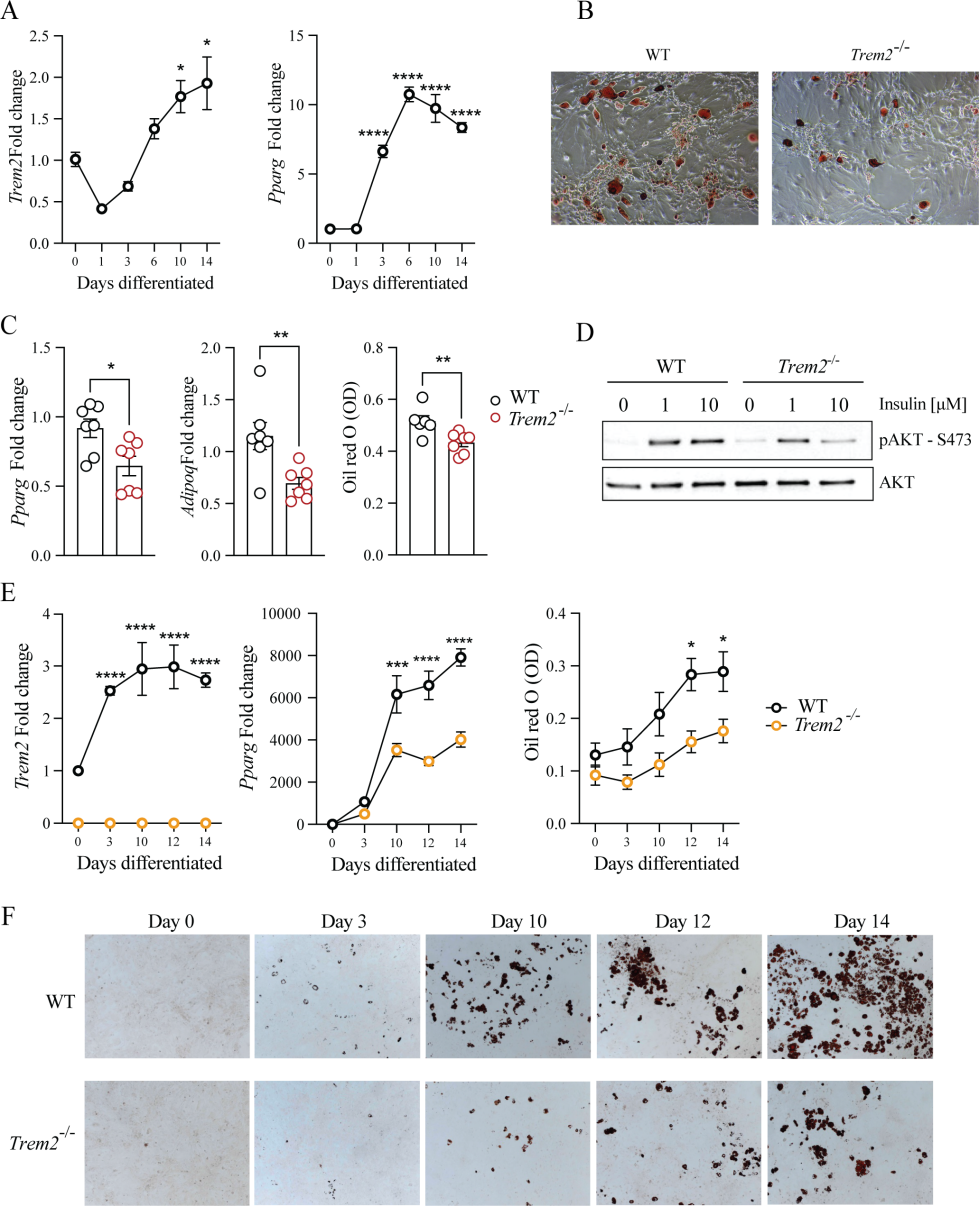
TREM2 promotes adipocyte differentiation. **(A)** Mouse *Trem2* and *Pparg* expression during adipocyte differentiation of stromal vascular fraction cells derived from epididymal white adipose (eWAT), n = 4 per genotype. **(B)** Representative oil red O staining of adipocytes derived from eWAT 14 days post-differentiation. Magnification is 50 x **(C)** *Pparg*, *Adipoq* and Oil Red O quantification in adipocytes derived from eWAT 14 days post-differentiation, n = 6–7 per genotype. **(D)** AKT signaling in WT and *Trem2*^-/-^ 14-day differentiated adipocytes derived from eWAT, post 20-minute insulin treatment **(E)** *Trem2*, *Pparg* and Oil Red O levels in adipocytes derived from mouse embryonic fibroblasts (MEF), n = 4 per genotype. **(F)** Representative oil Red O staining from **(E)**. Magnification is 5 x. Results in **(A, C, E)** represent mean ± SEM. All data is representative of 2 independent experiments except data in **(C)** which is pooled from 2 experiments. Statistical analysis was performed with one-way ANOVA followed by Tukey post-test **(A, E)** or Student’s T-test **(C)**. *P < 0.05, **P < 0.01, ***P < 0.001, ****P < 0.0001.

To examine a role for TREM2 in adipocyte differentiation, we next isolated murine SVF and differentiated the progenitors therein into mature adipocytes with an adipogenic cocktail. This demonstrated that *Trem2* was upregulated during adipogenesis, which coincided with pro-adipogenic *Pparg* expression (**Figure 2A**). Further, adipocytes derived from murine SVF of mice globally devoid of *Trem2* (*Trem2^−/−^* mice) displayed attenuated adipocyte differentiation and lipid acquisition as assessed using *Pparg*, *Adipoq* and oil red O staining (**Figures 2B, C**). As insulin promotes adipocyte differentiation and lipid metabolism via PI3K/AKT signaling (8, 26, 27), we next assessed AKT signaling in differentiated adipocytes from both genotypes, observing attenuated insulin-stimulated AKT signaling in *Trem2^−/−^* adipocytes (**Figure 2D**, **Supplementary Figure 2**). A similar impaired adipocyte differentiation was observed in *Trem2^−/−^* pre-adipocytes derived from mouse embryonic fibroblasts, and this was already evident at day 10 post differentiation (**Figures 2E, F**). Together, these data indicate TREM2 promotes adipocyte differentiation.

### 2.3 TREM2 deletion in PDGFR-α^+^ pre-adipocytes impacts early adipocyte differentiation

**Figure 3:**
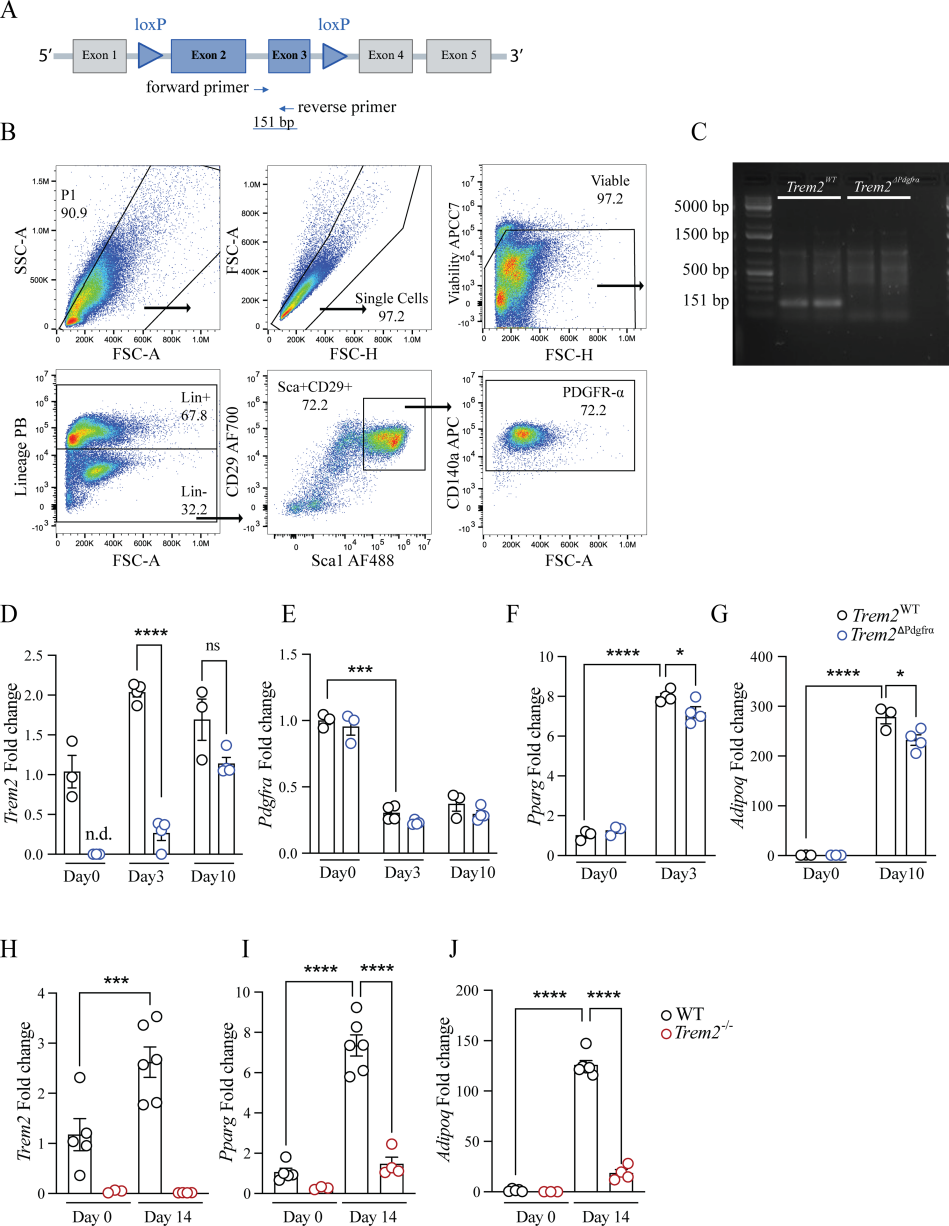
TREM2 deletion in PDGFR-α^+^ pre-adipocytes promotes early adipocyte differentiation. **(A)** Schematic representation of the *Trem2* floxed allele used to generate *Trem2* ^ΔPdgrfα^ animals. LoxP sites flank exons 2 and 3 of the *Trem2* gene. The locations of the forward and reverse primers used to confirm *Trem2* deletion are indicated and result in the loss of a 151bp PCR product. **(B)** Sorting strategy for PDGFR-α+ ASC. ASC are gated as single cells, viable, Lin^-^ (CD45^-^CD31^-^Ter119^-^CD11b^-^) cells. Cells were further gated as CD29^+^Sca1^+^PDGFR-α^+^. **(C)** Gel depicting deletion efficiency as determined using PCR on genomic DNA isolated from sorted Lin^-^CD29^+^Sca-1^+^PDGFR-α^+^ eWAT ASC from both genotypes of animals. **(D, E)** *Trem2* and *Pdgfra* levels in adipocytes derived from sorted Lin^-^CD29^+^Sca-1^+^PDGFR-α^+^ eWAT ASC from both genotypes of animals for the indicated time points, n = 3–4 per genotype. **(F)** *Pparg* levels during early adipocyte differentiation in adipocytes derived from sorted Lin^-^CD29^+^Sca-1^+^PDGFR-α^+^ eWAT ASC from both genotypes of animals, n = 3–4 per genotype. **(G)** *Adipoq* levels during late adipocyte differentiation in adipocytes derived from sorted Lin^-^CD29^+^Sca-1^+^PDGFR-α^+^ eWAT ASC from both genotypes of animals, n = 3–4 per genotype. **(H-J)** *Trem2*, *Pparg* and *Adipoq* expression in differentiated adipocytes derived from sorted Lin^-^CD29^+^Sca-1^+^PDGFR-α^+^ eWAT ASC from *Trem2* ^-/-^ mice, n = 3–6 per genotype. Data in **(D–J)** represent mean ± SEM and data in **(H–J)** is representative of 2 independent experiments. Statistical analysis was performed with Two-way ANOVA followed by Bonferroni post-test. *P < 0.05, ***P < 0.001, ****P < 0.0001.

Formally examining a role for ASC expressed TREM2 therein, we next crossed *Trem2*^fl/fl^ mice harboring loxP sites flanking exons 2 and 3 of the *Trem2* gene with *Pdgfra*-Cre expressing animals, generating *Trem2*^ΔPdgrfα^ animals (**Figure 3A**). We confirmed deletion by PCR on genomic DNA using specific primers that generate a 151bp product corresponding to exon 3 from isolated sorted Lin^-^CD29^+^Sca1^+^PDGFR-α^+^ ASC (**Figures 3B, C**). Subsequently, we differentiated Lin^-^CD29^+^Sca1^+^PDGFR-α^+^ ASC from both genotypes, observing that already at 3 days post-differentiation as *Pdgfra* decreased, *Trem2* increased. Confirming deletion efficiency, *Trem2* was not detectable at baseline in ASC derived from *Trem2*^ΔPdgrfα^ animals and was significantly decreased day 3 post-differentiation (**Figures 3D, E**). Consistent with *Pdgfra* downregulation during adipocyte differentiation, *Trem2* levels were comparable between genotypes in mature adipocytes at day 10 (**Figure 3D**). Demonstrating Cre specificity, while *Trem2* was deleted in PDGFR-α^+^ ASC, its levels were comparable between bone marrow derived macrophages (BMDM) isolated from both genotypes of animals (**Supplementary Figure 3A**). Adipocytes derived from *Trem2*^ΔPdgrfα^ ASC displayed moderate defects in early differentiation as *Pparg* levels were significantly decreased day 3 post-differentiation, however, we noted no differences when they were fully differentiated at day 10 (**Figures 3F**, **Supplementary Figure S3B**). Nonetheless, indicating that ASC from *Trem2*^ΔPdgrfα^ animals display defects in adipocyte maturation, although *Adipoq* levels were unchanged at day 3 they were significantly lower day 10 post differentiation (**Figure 3G**). We next examined adipocyte differentiation efficiency in sorted Lin^-^CD29^+^Sca1^+^PDGFR-α^+^ ASC from mice globally devoid of *Trem2*. This demonstrated *Trem2* levels were strongly attenuated late in differentiation and this was associated with profound downregulations in *Pparg* and *Adipoq* (**Figures 3H–J**). Together, these data indicate that TREM2 deletion in PDGFR-α^+^ pre-adipocytes is important for early adipocyte differentiation and reveal differences in differentiation efficiency between ASC derived from *Trem2*^ΔPdgrfα^ versus *Trem2^−/−^* animals.

### 2.4 TREM2 deletion in PDGFR-α^+^ pre-adipocytes moderately increases pre-adipocyte hyperplasia but has no effect on adipose remodeling in obesity

**Figure 4:**
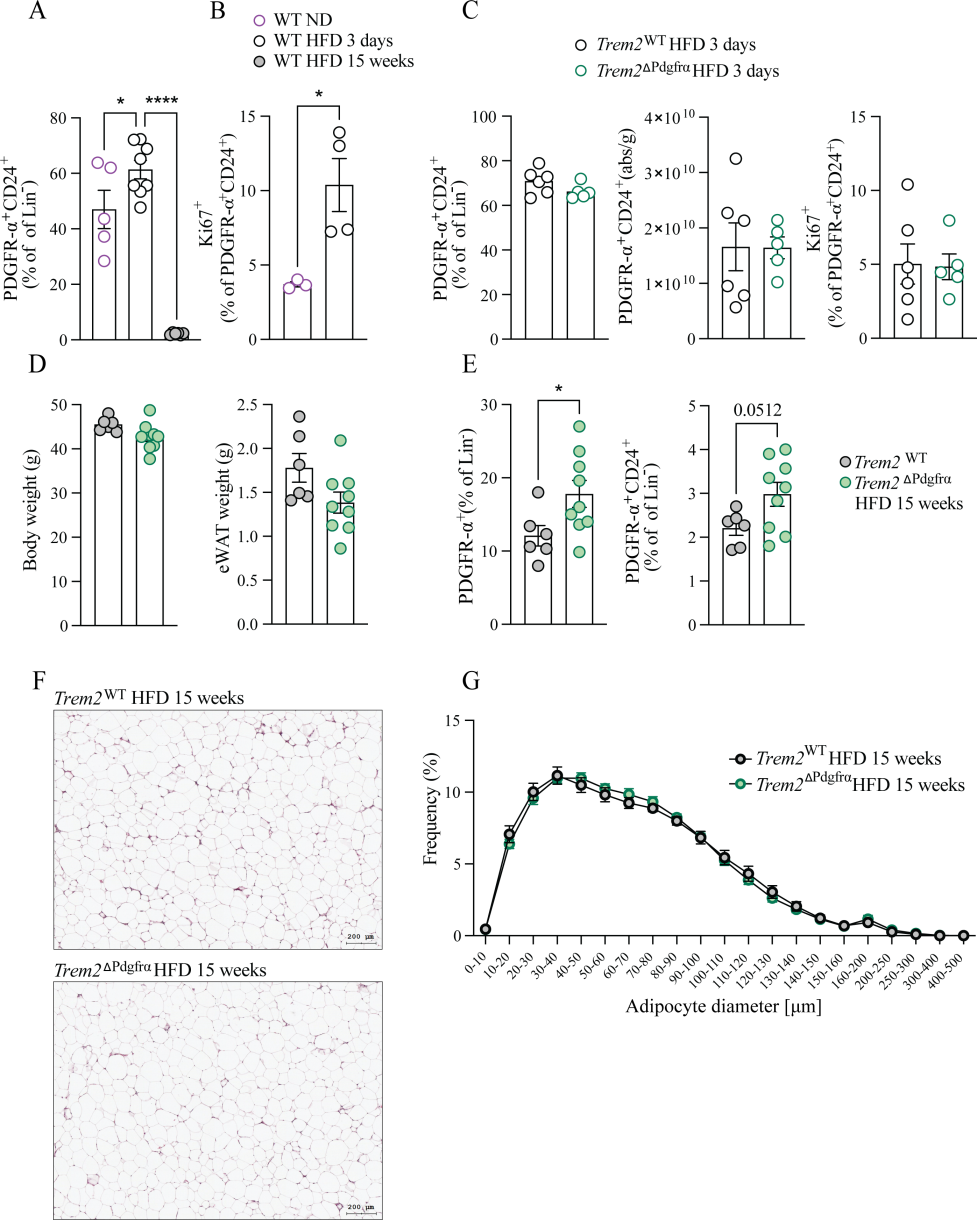
TREM2 expression in PDGFR-α^+^ pre-adipocytes moderately restrains pre-adipocyte hyperplasia but has no effect on adipose remodeling in obesity. **(A)** Percentage of PDGFR-α^+^CD24^+^ ASC within the lineage-negative pool of eWAT at the indicated times of HFD feeding. n=5-8. **(B)** Percentage of Ki67^+^ PDGFR-α^+^ CD24^+^ ASC three days post HFD. n = 3-4. **(C)** Percentage of PDGFR-α^+^CD24^+^ ASC within the lineage negative pool of eWAT (left), absolute numbers (middle) and percentage Ki67^+^ (right) therein in both genotypes 3 days post HFD. n=5-6. **(D)** Body and eWAT weight of *Trem2* ^ΔPdgrfα^ animals and controls 15 weeks post HFD. n=6-9. **(E)** Percentage of PDGFR-α^+^ or PDGFR-α^+^CD24^+^ ASC within the lineage negative pool of eWAT of *Trem2* ^ΔPdgrfα^ and littermate control animals 15 weeks post HFD. n=6-9 **(F)** Representative H&E staining of eWAT in both groups of animals post 15 weeks HFD. **(G)** Quantification of adipocyte frequency from **(F)**. n = 13–16 mice per genotype. All data represent mean ± SEM and are representative of 2 independent experiments. Data in A and G is pooled data from 2 independent experiments. Statistical analysis was performed with one-way ANOVA followed by Dunnett post-test **(A)** or Student’s T-test **(B–G)**. *P < 0.05, ***P < 0.001, ****P < 0.0001.

We next assessed how PDGFR-α-expressing ASC proliferate during obesity. Consistent with observations that ASC proliferation peaks within the first week of HFD feeding (6), flow cytometry analysis revealed that Lin^-^CD29^+^Sca1^+^PDGFR-α+CD24^+^ ASC within the eWAT increased three days post HFD and were dramatically reduced 15 weeks post HFD, at which time they constituted around 2% of the ASC pool (**Figure 4A**, **Supplementary Figure 4**). Intracellular Ki67 staining confirmed active proliferation within Lin^-^CD29^+^Sca1^+^PDGFR-α^+^CD24^+^ ASC occurred three days post HFD (**Figure 4B**, **Supplementary Figure 4**). Together, these data indicate adipocyte hyperplasia occurs early in obesity and that late in obesity, the PDGFR-α^+^ ASC pool is diminished. Subsequently, we placed both genotypes on a HFD for 3 days, observing comparable proliferation, ASC percentages and absolute ASC numbers within the lineage-negative eWAT pool between genotypes (**Figure 4C**). Noting that Lin^-^CD29^+^Sca1^+^PDGFR- α ^+^CD24^+^ ASC are diminished late in obesity (**Figure 4A**), we next fed *Trem2*^ΔPdgrfα^ animals and their littermate controls a HFD for 15 weeks, observing comparable body and eWAT weight between genotypes (**Figure 4D**). At this time point, relative to controls, *Trem2*^ΔPdgrfα^ animals exhibited a strong albeit statistically insignificant tendency (p=0.0512) towards elevated Lin^-^CD29^+^Sca1^+^PDGFR-α^+^CD24^+^ ASC within their eWAT, which was likely due to significantly increased percentages of PDGFR-α^+^ cells in the Lin^-^ pool (**Figure 4E**). This was however not associated with any impacts on mature adipocyte morphology or size as histological analysis indicated comparable adipocyte frequencies between genotypes (**Figures 4F, G**). Together, although late in obesity *Trem2*^ΔPdgrfα^ animals exhibit moderately elevated hyperplasia, TREM2 deletion in pre-adipocytes ultimately exerts no effects on adipose remodeling.

### 2.5 TREM2 deletion in pre-adipocytes exerts no influence on metabolic health or ATMs

**Figure 5:**
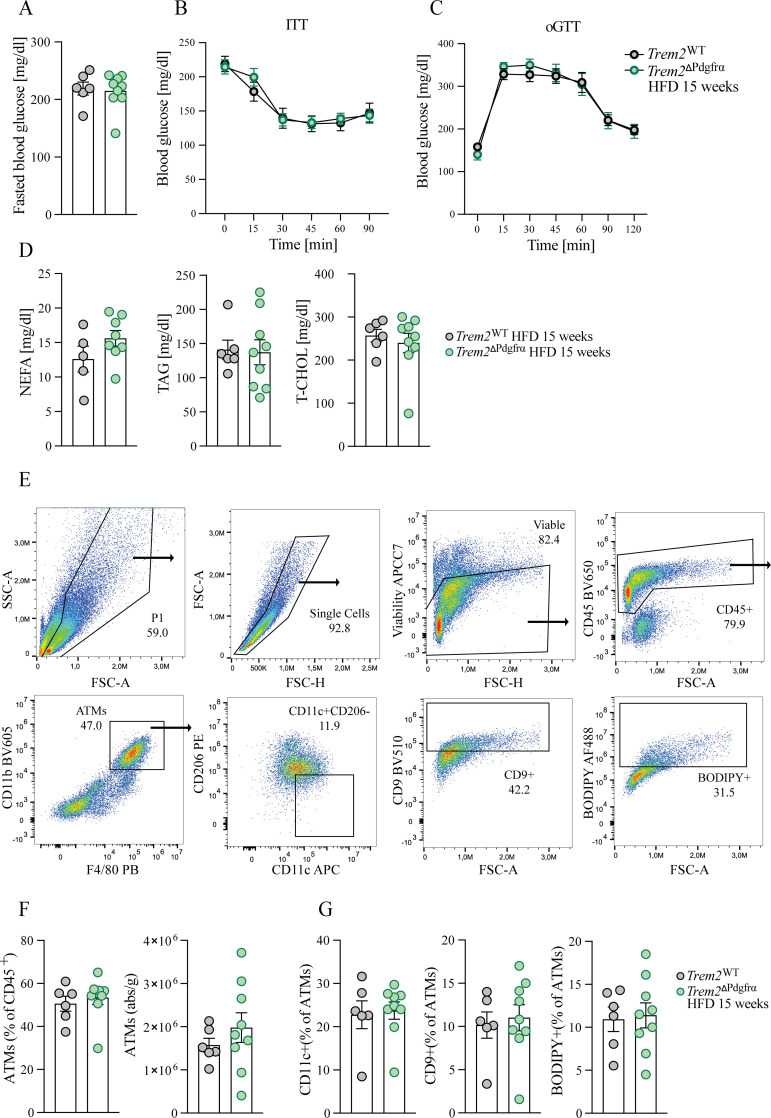
No influence of TREM2 deletion in PDGFR-α^+^ cells on metabolic health and ATMs. **(A)** Fasted blood glucose of *Trem2* ^ΔPdgrfα^ animals and controls 15 weeks post HFD. n=6-9. **(B, C)** Insulin tolerance (ITT) **(B)** or oral glucose tolerance test (oGTT) **(C)** of *Trem2* ^ΔPdgrfα^ animals and controls 15 weeks post HFD. n=6-9. **(D)** Serum non-esterified fatty acids (NEFA), triglyceride (TAG), and total cholesterol (T-CHOL) in *Trem2* ^ΔPdgrfα^ animals and controls 15 weeks post HFD. n=5-8. **(E)** Gating strategy used to identify ATM populations. ATMs are gated as single cells, viable, CD45^+^CD11b^+^F4/80^+^ cells. Within the ATM pool the cells were further gated as CD11c^+^CD206^-^ or CD9^+^ or BODIPY^+^. **(F)** Percentage of CD11b^+^F4/80^+^ ATMs within the CD45^+^ immune cell pool and absolute macrophage amounts in both genotypes of obese animals, 15 weeks post HFD. n=6-9. **(G)** Percentage of CD11c^+^, CD9^+^ or BODIPY^+^ macrophages within the CD11b^+^F4/80^+^ ATM pool in *Trem2* ^ΔPdgrfα^ animals and controls 15 weeks post HFD. n=6-9. All data represent mean ± SEM and are representative of 2 independent experiments.

We next examined the influence of ASC-expressed TREM2 on metabolic health and ATM amounts. Indicating TREM2 exerted no impacts therein, basal fasted glucose levels as well as insulin and glucose tolerance were identical between obese genotypes post 15 weeks HFD (**Figures 5A–C**). Further, there were no differences in serum non-esterified free fatty acids (NEFAs), triglycerides or cholesterol (**Figure 5D**). Evaluating CD11b^+^F4/80^+^ ATMs in eWAT revealed no differences in ATM amounts within the CD45^+^ immune cell pool. Moreover, absolute ATM numbers were similar between obese genotypes (**Figures 5E, F**). Examining ATM subtypes indicated that TREM2 deficiency within ASC did not impact metabolically detrimental circulation-derived CD11b^+^F4/80^+^CD11c^+^ ATM (9, 12). Moreover, lipid-laden ATMs were identical as the percentage of CD9-expressing and BODIPY^+^ ATMs (18, 20) were similar in both genotypes of obese eWAT (**Figures 5E, G**).

## 3 Discussion

How WAT remodels in obesity directly influences metabolic disease risk. When lipids are excessively stored in mature adipocytes without hyperplasia this leads to adipocyte dysfunction characterized by adipose hypertrophy and macrophage accumulation. Thus, failures in adipose tissue plasticity are associated with worsened metabolic health (1, 3, 28).

While a complete lack of adipogenesis is linked to lipodystrophies and severe metabolic complications (29), healthy fat expansion via adipogenesis improves metabolic health despite increased body mass (30–32). ASC from mice globally devoid of TREM2 strongly promoted adipogenesis in 3 different experimental set-ups including sorted PDGFR-α^+^ ASC, with *Trem2* being absent in *Trem2*^-/-^ cells during the examined differentiation time course. Conversely, and in line with *Pdgfra* downregulation during adipocyte differentiation, *Trem2*^ΔPdgrfα^ ASC displayed decreased *Pparg* expression only during early differentiation, indicating TREM2 is important for adipocyte commitment. In this experimental set-up, we noted comparable *Trem2* levels between genotypes 10 days post-differentiation. Together, these data suggest a late-stage, cell autonomous role for TREM2 on mature adipocytes differentiated from ASC of mice globally devoid of TREM2 that is absent in the conditional TREM2 deletion experimental set up. Thus, *Trem2* expression on ASC as well as mature adipocytes is crucial for adipogenesis.

*Trem2*^ΔPdgrfα^ animals exhibited moderately increased PDGFR-α^+^ ASC progenitors late in obesity but no differences in adipose hypertrophy or metabolic health compared to controls. While PDGFR-α^+^ ASC definitely contribute to adipocyte turnover within WAT, the PDGFR- α cre has limitations. This includes its broad targeting of mesenchymal subsets in WAT and other organs, including WAT fibrogenic-inflammatory progenitors or skeletal muscle FAPs, which we acknowledge could lead to confounding effects during HFD feeding. Further, as ASC are extremely heterogeneous, other ASC subtypes including *Pdgfrb*, *Dpp4*, or *Icam1* expressing ASC could compensate for the deficiency of TREM2 within the PDGFR-α^+^ ASC pool (1–5).

Although ASC populations have not been studied in mice globally devoid of TREM2, *Trem2*^-/-^ animals exhibit augmented adipose hypertrophy and worsened metabolic health post 15 weeks HFD feeding (18–20). Animals fed HFD exhibit increased amounts of TREM2^+^CD9^+^ lipid-associated macrophages (LAM) within their visceral WAT that are found in close proximity to dying adipocytes in crown-like structures (18). Bone marrow transplantation experiments utilizing *Trem*2^-/-^ bone marrow have revealed conflicting results on the role of macrophage expressed TREM2 in metabolic health. One study suggested the worsened metabolic health of obese *Trem*2^-/-^ animals is mediated by hematopoietic/macrophage expressed TREM2. However, our own previous work indicates it is related to WAT intrinsic mechanisms that are uncoupled from the ability of hematopoietic/macrophage expressed TREM2 to restrain adipose remodeling (18, 20). Regardless both these studies demonstrated decreased ATM amounts and lipid-ladenness in eWAT of obese *Trem*2^-/-^ animals, while the *Trem2*^ΔPdgrfα^ animals examined in this study exhibited no differences in these parameters. Further, obese animals lacking exon 2 of TREM2 display no metabolic phenotype but exhibit elevated adipose hypertrophy and CD11b^+^ ATMs from these mice exhibit attenuated expression of LAM genes (33). Together, although TREM2 expression within LAMs is likely important for the clearance of lipids and dying adipocytes (18, 34) and might hence be critical for adipose remodeling, the exact role of macrophage expressed *Trem2* in metabolic health requires further work. Looking forward, generating conditional TREM2-deficient mice in mature adipocytes and macrophages will undoubtedly yield further insight into how TREM2 impacts WAT biology and metabolic health.

## 4 Methods

### 4.1 Experimental animals

All procedures were conducted in compliance with protocols approved by the Medical University of Vienna and the Austrian Ministry of Sciences under the project numbers: BMWF-66.009/0276-II/3b/2013 and 2024-0.728.389. *Trem2^−/−^* mice containing a deletion in exons 3 and 4 and backcrossed onto a 98% C57BL/6 background were generated as previously described (35). For the generation of conditional TREM2 knockout mice, Trem2^tm1c(EUCOMM)Wtsi mice (#029853, Jackson Laboratory) carrying floxed alleles of *Trem2* were crossed with C57BL/6-Tg(Pdgfrα-cre)1Clc/J mice (#013148, Jackson Laboratory), which express Cre recombinase under the control of the *Pdgfrα* promoter to achieve adipocyte progenitor–specific *Trem2* deletion. The resulting mice are hereafter referred to as *Trem2*^ΔPdgfrα^ mice, and their Cre-negative littermates as *Trem2*^WT^ controls. Genotyping primers used on toe clip biopsies were: *Trem2* primer forward 5’-CAAAGGAAGGGTCCATTTTG-3’, reverse, 5’-CCTGACCTATGGGCAGTAACA-3’. Band size is 312 bp. Cre primer forward 5’-CTCCTCTACTCCATTCTTCCC-3’, reverse, 5’-ACTCCCACCAATGAACAAAC-3’. Band size for the Cre primer is 455 bp.

All mice were bred at the Medical University of Vienna and housed under SPF conditions with temperatures ranging from 21-23°C in cages (4 mice per cage) with micro-isolator tops with a 12-hour light cycle (7 am – 7 pm). For all experiments except those involving dietary intervention, littermates matched male mice (8–12 weeks old) were used. All mice were maintained on a standard chow diet (#V1536, Ssniff). For dietary intervention studies, feeding began at 6 weeks of age with a high-fat diet providing 60% of calories from fat (#D12492, Research Diets).

### 4.2 Glucose and insulin tolerance tests

After an overnight fasting period, mice received an oral gavage of 20% glucose solution (1 g/kg), and blood glucose levels were determined from tail vein samples at the specified time points. Insulin sensitivity was evaluated following a 2-hour fast through intraperitoneal injection of human regular insulin (0.75 U/kg). Blood glucose concentrations were measured using an Accu-Check glucometer together with Accu-Check Instant Test Strips (#07819382, Accu-Check). Mice were given 4–6 days to recover after the insulin tolerance test before performing the oral glucose tolerance assay.

### 4.3 Mouse laboratory parameters

Triglycerides (#F1520, Lab Technologies Medizintechnik GmbH) and total cholesterol (#F1519, Lab Technologies Medizintechnik GmbH) were quantified using the Fuji Dri-Chem NX600 V analyzer (#F16485, Fujifilm), and non-esterified free fatty acids (NEFA) were measured using the NEFA-HR (2) R1 and R2 set (#436-91995, #434-91795, FUJIFILM Wako Chemicals Europe GmbH).

### 4.4 Stromal vascular fraction isolation

Stromal vascular fraction (SVF) was isolated as previously described (20, 36). Epididymal white adipose fat pads were removed and cut into small pieces (approximately 2mm) and washed 2 x with DMEM:F12 media (#11320-074, Gibco) supplemented with 1% penicillin–streptomycin (#D5796, Sigma-Aldrich) by centrifugation for 6 min at 1250 rpm at room temperature. Adipose was digested in 10 ml of freshly prepared collagenase II solution (1 mg/ml in 1.5% sterile BSA, in DMEM:F12/penicillin–streptomycin) and the fat allowed to digest in a shaker at 37°C for 45 min. Subsequently, the digested adipose-enzyme solution was filtered through a 70 μm strainer into 10 ml DMEM:F12 media supplemented with 10% FBS, 1% penicillin–streptomycin (herein referred to as SVF media), and centrifuged at 1250rpm (5 min, room temperature). Post centrifugation, the pellet was resuspended in SVF media, washed twice, and resuspended in 1ml of erythrocyte lysis buffer for 5 min, after which 15 ml of SVF media was added, cells were centrifuged, and the pellet was resuspended in 1 ml SVF media. The cells were then cultured in full SVF media in T-75 flasks at 37°C, 5% CO_2,_ until 80% confluency was reached.

### 4.5 Labelling, flow cytometry and asc isolation

ASC were isolated from SVF cells using FACS sorting as previously described (6, 7). Isolated SVF was resuspended in PBS supplemented with 2% FBS for antibody labeling and stained with an antibody cocktail containing: Fixable Viability Dye APCC7 780 (#13539140, Invitrogen, 1:1000), Ter119 PB (#B116231, BioLegend, 1:200), CD31 PB (#B102423, BioLegend, 1:50), CD45 PB (#B109820, BioLegend, 1:200), CD11b PB (#10140233, Invitrogen, 1:100), Sca-1 AF488 (#B108133, BioLegend, 1:200), CD29 AF700 (#B102218, BioLegend, 1:100), CD140a APC (#B135907, BioLegend, 1:30) for 30 min at 4°C. Cells were subsequently centrifuged at 1250 rpm (5 min, RT). Control staining included single staining and unstained cells. ASC were identified as live, Lin^-^(CD45^-^CD31^-^Ter119^-^CD11b^-^) CD29^+^Sca1^+^PDGFR-α^+^ cells and sorted using flow cytometry with a BD Aria Fusion cell sorter. For intracellular staining identifying ASC proliferation, ASC were stained as above, with an additional permeabilization step using the Intracellular Fixation & Permeabilization Buffer Set and the Foxp3/Transcription Factor in combination with Ki67 PE staining (#12-5698-82, Thermo Fisher Scientific) or isotype control (#12-4321-80, Thermo Fisher Scientific). Cells were acquired with CytoFlexS Flow cytometer (Beckman Coulter) and data further analyzed with FlowJo software (v.10.8.1).

### 4.6 Generation of mouse embryonic fibroblasts

Embryos were retrieved from pregnant WT and *Trem2^−/−^* females 12.5 days postcoitum. The brain and internal organs of the embryos were removed, and the remaining carcasses were minced into fine and homogenous pieces with MEF media (DMEM supplemented with 10% FBS/1% Penicillin/Streptomycin). The resulting cell suspension was placed into a new 10 cm dish overnight at 37°C 5% CO_2_. Subsequently, red blood cells were removed, and fresh MEF media was added. 48 hr post-isolation, cells were harvested using trypsin and split into a 15 cm tissue culture dish. When 100% confluence was reached, cells were frozen in liquid nitrogen and thawed when needed. In all studies, primary MEFs (passage 3-8) were used.

### 4.7 Adipocyte differentiation

ASC, SVF, or MEF were allowed to reach confluency in 24-well tissue culture plates before differentiation. Differentiation was started by changing the respective cell culture media DMEM:F12/10% FBS/1% penicillin–streptomycin for ASC, SVF, or MEFs to media containing 5 µg/ml Insulin, 5 µM Troglitazone, 0.5 mM Isobutylmethylxanthine, and 1 µM Dexamethasone (#I9278, #T2573, #I5879, and #D8893, Sigma-Aldrich). After 48 hr, Isobutylmethylxanthine and Dexamethasone were removed from the media, and cells were solely supplied with media supplemented with 5 µg/ml Insulin and 5 µM Troglitazone, which was exchanged every 2–3 days.

### 4.8 Bone marrow–derived macrophage culture

Bone marrow cells were flushed from murine femurs and tibias and cultured in full RPMI medium supplemented with 10 ng/mL macrophage colony-stimulating factor (M-CSF) to induce macrophage differentiation. Cells were maintained under standard conditions (37°C, 5% CO_2_) for 6 days. On day 6, adherent macrophages were detached using Cellstripper (#25-056-CI, Corning) for 10 minutes at 37°C. Cells were reseeded at 5 × 10^5^ cells per well in medium containing M-CSF and harvested the following day for downstream analyses.

### 4.9 Oil red O staining of adipocytes & quantification

Formalin fixated adipocytes in 24-well tissue culture dishes were washed with 1 ml of 60% isopropanol and dried completely. 200 µl of Oil Red O (#O-0625, Sigma-Aldrich) working solution (composed of 4 parts water and 6 parts 0.6% Oil Red O dye in isopropanol) was added for 10 min, after which the wells were washed 4 times with H_2_O. At the last wash, H_2_O was left in the wells so that microscopy could be performed. For quantification, water was removed, and following complete drying, 750 µl of 100% isopropanol was added to the wells. 10 min post-incubation, Oil Red O content was measured using a plate reader at 450 nm.

### 4.10 Adipocyte cell size quantification

Adipose tissues were fixed in 4% formaldehyde overnight and subsequently embedded in paraffin following dehydration in a 96% ethanol series. Sections of 5 µm thickness were stained with hematoxylin and eosin (H&E). The slides were scanned using a TissueGnostics Slide Scanner, and image analysis was performed with the StrataQuest-based Adipocyte App to automatically quantify adipocyte number, cell size, and cell diameter within the analyzed fat tissue.

### 4.11 Western blotting

Proteins were extracted following adipocyte differentiation using ice cold RIPA buffer (1% Triton X-100, 0.1% SDS, 0.5% Deoxycholic acid, 150 mM NaCl, 50 mM Tris-HCl, pH 7.5) containing protease inhibitors (#11836153001, Complete Mini, Roche used at 1:50), 10 mM NaF, 1 mM Na_3_VO_4_ (#450243, Sigma-Aldrich) and 0.250 U/µl Benzonase Nuclease HC (#71204, Novagen). The homogenate was cleared by centrifugation at 4°C for 5 min at 14,000 rpm, and protein content in the supernatant fraction was determined using the Pierce Protein Assay Kit (#23225, Thermo Scientific). 20 μg of proteins were resolved by SDS-PAGE and transferred to PVDF membranes (Biorad). Membranes were blocked with 5% BSA in PBS containing 0.05% Tween-20 (PBS-T) and incubated with primary antibodies at 4°C overnight at a concentration of 1:1000. The following antibodies were used: Akt Pan (#4691, Cell Signaling), Phospho-Akt Ser473 (#4060, Cell Signaling) and β-actin (#A5441, Sigma-Aldrich). Subsequently, membranes were washed 3 times for 10 min with PBST before incubation with the appropriate anti-IgG-horseradish peroxidase-linked (HRP) secondary antibody for 1 hr at room temperature - anti-rabbit IgG (#7074, Cell Signaling, used at 1:1000 in 5% BSA/PBST) or goat anti-mouse IgG (#1706516, Biorad, used at 1:1000 in 5% BSA/PBST). Thereafter, membranes were washed again 3x 10 min with PBST and the desired protein was detected using enhanced chemiluminescence.

### 4.12 Quantitative real time PCR

qRT-PCR was performed as described (37). In brief, total RNA was extracted from cells using TRIzol according to the manufacturer’s instructions (#15596-018, Ambion). For RNA purification and cDNA synthesis, equal amounts of RNA (up to 1μg) were resuspended in 13μl H_2_O and 1.5μl 10x buffer plus 0.5μl DNase I taken from the PerfeCta Dnase I Kit (#95150-01K, Quanta Biosciences) for DNA digestion. Samples were incubated at 37°C for 30 min before 1.5μl Stop solution was added and incubated thereafter at 65°C for another 10 min. Reverse transcription into cDNA was conducted using the iScript cDNA Synthesis Kit according to the manufacturer’s instructions (#170-8891, Bio-Rad). qRT-PCR was performed using the iTaq SYBR Green Supermix with ROX according to the supplier (# 72-5853, Bio-Rad). PCR was performed with the StepOnePlus™ Real-Time PCR System (Applied Biosystems). Post amplification melting curve analysis was performed to check for unspecific products and primer-only controls were included to ensure the absence of primer dimers as well as H_2_O only controls to check for contamination by genomic DNA. For normalization threshold cycles (Ct-values) were normalized to the housekeepers, Hypoxanthine Guanine Phosphoribosyltransferase (*Hprt*) within each sample to obtain sample-specific ΔCt values (= Ct_gene of interest_– Ct_housekeeper_). 2−ΔΔCt values were calculated to obtain fold expression levels, where ΔΔCt = (ΔCt_treatment_-ΔCt_control_). Primer sequences are indicated in **Supplementary Table 1**.

### 4.13 Genomic DNA isolation

ASC were sorted as above. Genomic DNA was extracted according to the manufacturer’s instructions using the DNeasy Blood & Tissue Kit (#69504, QIAGEN GmbH). *Trem2* deletion was confirmed in DNA from isolated ASC using primers spanning exon 3. *Trem2* primer forward 5’-TACACAAGACTGGAGCCCTG-3’, reverse, 5’- ACCCAGAGATCTCCAGCA TC-3’. Band size for WT is 151 bp for *Trem2*^WT^ ASC which is absent in *Trem2*^ΔPdgfrα^ ASC.

### 4.14 Sc-RNA and sn-RNA seq data analysis

Two datasets encompassing sc-RNAseq and sn-RNAseq were analyzed (24, 25), using statistical software R (version 4.4.2) and package Seurat (38) (version 5.4.0). In both cases we used the final Seurat objects post quality control, including t-SNE and UMAP projections, used for analysis in the respective publications. While we used the entire annotated objects for depicting all the cell subpopulations, for further analysis of pre-adipocytes we computationally excluded cells with one or more reads mapping to *Adgre1* or *Itgam* from the respective Seurat objects, keeping the original projections for plotting.

### 4.15 Statistical analysis

Data are expressed as mean ± SEM. All groups presented were normally distributed as determined using the Shapiro–Wilk and Kolmogorov–Smirnov tests. Sample sizes were based on previous experience with experiments powered to match sample sizes typical of the technique employed. Statistical significance in two-group comparisons was assessed with an unpaired Student’s t-test. ITT, oGTT and adipocyte differentiation data were analyzed using a two-way ANOVA followed by a Bonferroni correction with both time and group (i.e. genotype) as sources of variation. For multivariable comparisons, we performed a one-way ANOVA followed by a Dunnett or Tukey’s multiple comparison correction. Results were analyzed with GraphPad Prism software version 10, and a p < 0.05 was regarded as statistically significant.

## Data Availability

The raw data supporting the conclusions of this article will be made available by the authors, without undue reservation. The results published here are in part based on analysis of publicly available scRNA and snRNA-seq deposited under Gene Expression Omnibus (GEO), datasets GSE237143 and GSE160729.
