# Supplementary material for: TREM2 promotes adipogenesis of PDGFR-α+ adipose stem cells but is dispensable for adipose remodeling and metabolic health during diet-induced obesity
Source: Front Endocrinol (Lausanne). 2026 Apr 23;17:1738472. doi: 10.3389/fendo.2026.1738472 (PMC13151143; doi:10.3389/fendo.2026.1738472)
Supplement: Supplementary file 1 [file DataSheet1.pdf]

TREM2 promotes adipogenesis of PDGFR-α$^+$ adipose stem cells but is dispensable for adipose remodeling and metabolic health during diet-induced obesity

Anja Dobrijevic[1,2], Ana Korosec[1,2], Julia Stefanie Brunner[1,4], Lenka Matejovicova[1,2], Anna Gemza[1,2], Karin Lakovits[3], Hon Shing Lam[1,2], Aloña Agirre-Lizaso[1,2], Gernot Schabbauer[1] Sylvia Knapp[3] and Omar Sharif[1,2*]

[1]Institute of Vascular Biology and Thrombosis Research, Centre for Physiology and Pharmacology, Medical University of Vienna, Vienna, Austria.
[2]Christian Doppler Laboratory for Immunometabolism and Systems Biology of Obesity-Related Diseases (InSpiReD), Vienna, Austria.
[3]Research Division of Infection Biology, Department of Medicine I, Medical University of Vienna, Vienna, Austria.
[4]Present address: Cell Biology Program, Sloan Kettering Institute, Memorial Sloan Kettering Cancer Center, New York, NY, USA.

***Correspondence:**
Corresponding Author
omar.sharif@meduniwien.ac.at

Keywords: White adipose tissue, TREM2, Obesity, Adipose stem cell, Metabolic health, Adipogenesis

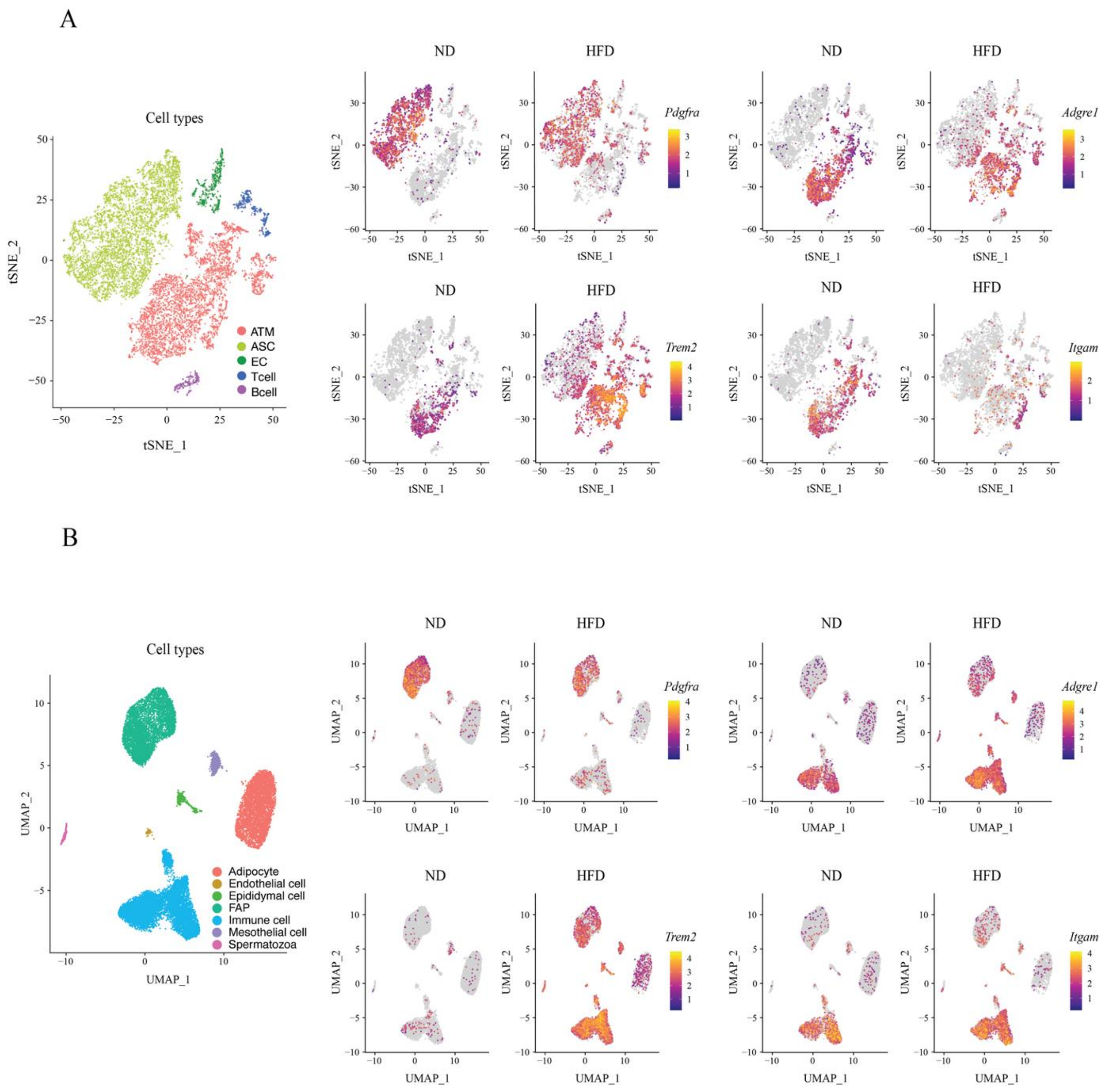

Fig. S1 Fidelity of clusters utilized for sc-RNA and sn-RNA seq analysis

(A) t-distributed stochastic neighbor embedding (t-SNE) plot of 14,205 SVF cells from eWAT of lean (18 weeks ND) and obese (12 weeks HFD) mice from Gene Expression Omnibus number GSE237143. The original clustering and cell annotations from this data set were kept, which identify 5 cell types. *Pdgra* and *Adgre1* or *Itgam* projection identity the ASC and ATM clusters, respectively. *Trem2* is additionally projected within the clusters with prominent expression in ATMs, but also some in ASC. **(B)** Uniform Manifold Approximation and Projection (UMAP) plot of FAP from eWAT of lean (18 weeks ND, 10227 cells) and obese (18 weeks HFD, 9498 cells) mice from Gene Expression Omnibus number GSE160729. The original clustering and cell annotations were retained, which identify 7 cell types. *Pdgra* and *Adgre1* or *Itgam* projection identity the FAP and ATM clusters, respectively. *Trem2* is additionally projected within the clusters with prominent expression in ATMs, but also in some in FAP.

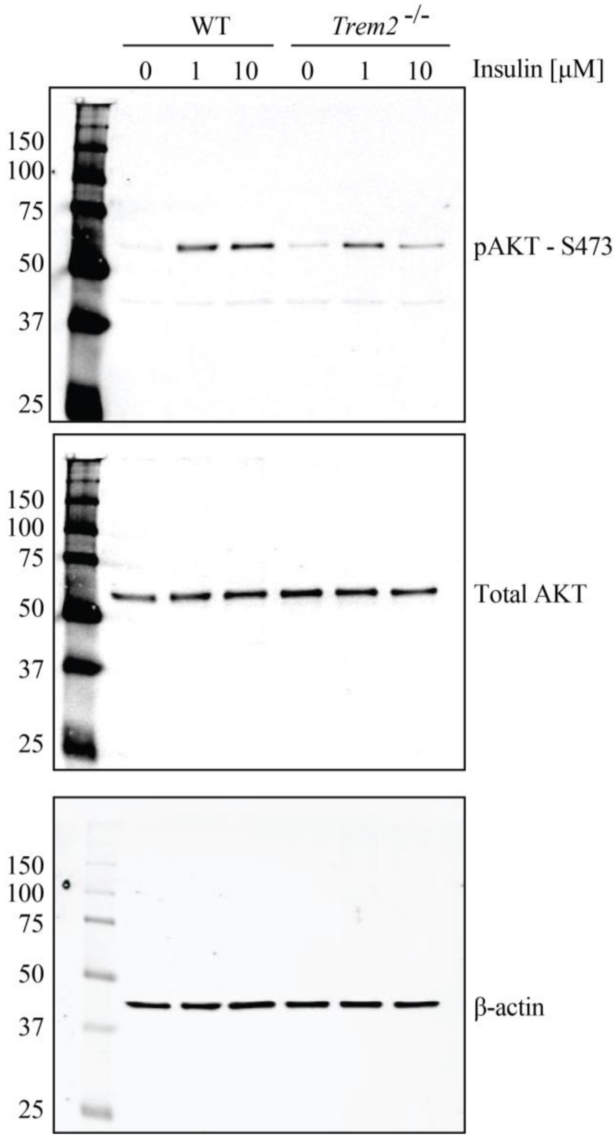

Fig. S2 Original uncropped scans of western blots for Fig. 2D

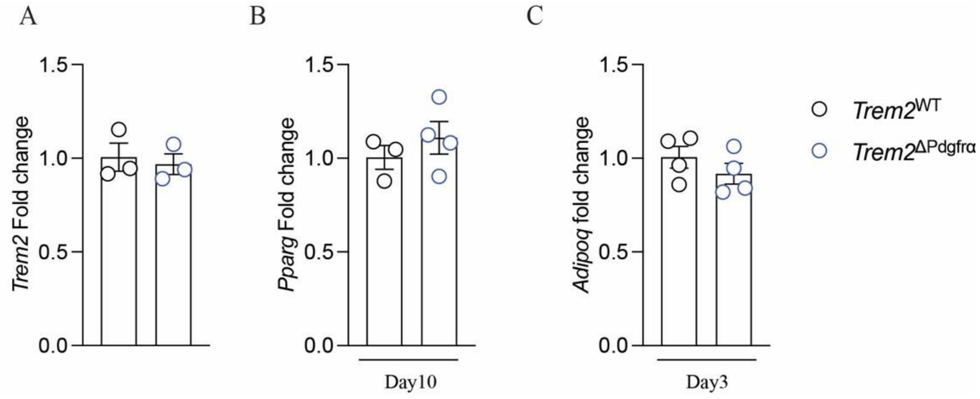

Fig. S3 Relative gene expression of *Trem2* in BMDMs and adipogenic markers in differentiated ASC

mRNA expression levels of *Trem2* in BMDMs (A) or *Pparg* (B) and *Adipoq* (C) at day 3 or 10 respectively of differentiated adipocytes derived from sorted Lin-CD29+Sca-1+PDGFR-α+ eWAT ASC, n = 3-4 per genotype are presented as fold change relative to control. Results represent mean ± SEM. Statistical analysis was performed with Student's T-test.

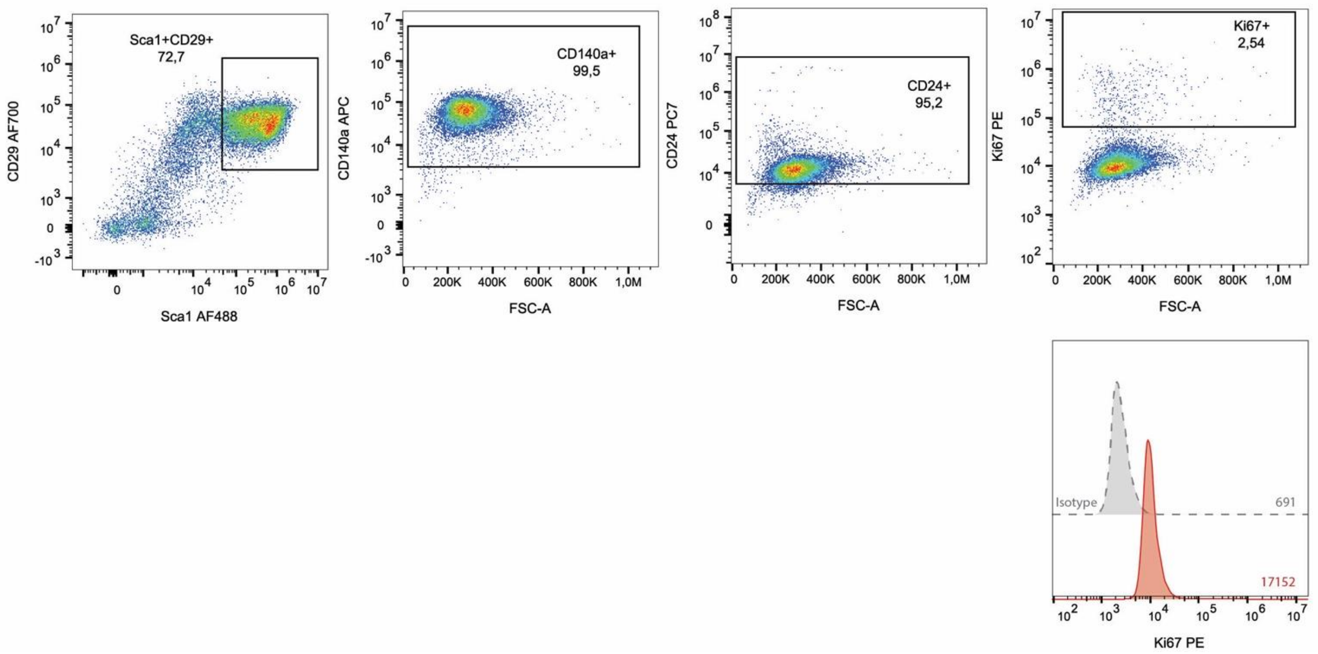

Fig. S4 Flow cytometry gating strategy for the identification of committed ASC and their proliferation.

ASC are pregated as single cells, viable, Lin^- ($CD45^-CD31^-Ter119^-CD11b^-$) cells. Cells were further gated as $CD29^+Sca1^+PDGFR\text{-}\alpha^+CD24^+$. Ki67 staining was used to examine proliferation within ASC and histograms depicting Ki67 or isotype control antibody are indicated.

Supplemental Table 1. RT-PCR Primers used in this study

Gene	Reference Sequence	Primer Sequence
	**Mouse**	
Adipoq	NM_009605.4	(Forward) AGCATCCTGAGCCCTTTTGGTGT<br>(Reverse) TAAGCTGGGGTCTGCCTGTCC
Hprt	NM_013556	(Forward) GTTAAGCAGTACAGCCCCAAAATG<br>(Reverse) AAATCCAACAAAGTCTGGCCTGTA
Trem-2	NM_031254	(Forward) CTGGCCTGCGTTCTCCTGA<br>(Reverse) GGTGGAGGAGGGGAGAGCAT
Pparg	NM_011146.3	(Forward) ACCCAGAGCATGGTGCCTTCGC<br>(Reverse) CCGAAGTTGGTGGGCCAGAATGGCA
Rplp0	NM_007475	(Forward) CTCTCGCTTTCTGGAGGGTG<br>(Reverse) ACGCGCTTGTACCCATTGAT
Trem2 floxed	NM_031254	(Forward) CTGGAACCGTCACCATCACTC<br>(Reverse) CGAAACTCGATGACTCCTCGG
